# Overexpression of Phosphate Transporter Gene *CmPht1;2* Facilitated Pi Uptake and Alternated the Metabolic Profiles of Chrysanthemum Under Phosphate Deficiency

**DOI:** 10.3389/fpls.2018.00686

**Published:** 2018-07-20

**Authors:** Chen Liu, Jiangshuo Su, Githeng’u K. Stephen, Haibin Wang, Aiping Song, Fadi Chen, Yiyong Zhu, Sumei Chen, Jiafu Jiang

**Affiliations:** ^1^College of Horticulture, Nanjing Agricultural University, Nanjing, China; ^2^Key Laboratory of Landscape Agriculture, Ministry of Agriculture, Nanjing, China; ^3^College of Resources and Environmental Sciences, Nanjing Agricultural University, Nanjing, China

**Keywords:** *Chrysanthemum morifolium*, *CmPht1;2*, phosphate transporter, phosphorus, metabolome

## Abstract

Low availability of phosphorus (P) in the soil is the principal limiting factor for the growth of cut chrysanthemum. Plant phosphate transporters (PTs) facilitate acquisition of inorganic phosphate (Pi) and its homeostasis within the plant. In the present study, *CmPht1;2* of the Pht1 family was cloned from chrysanthemum. CmPht1;2 is composed of 12 transmembrane domains and localized to the plasma membrane. Expression of *CmPht1;2* in roots was induced by Pi starvation. Chrysanthemum plants with overexpression of *CmPht1;2* (Oe) showed higher Pi uptake, as compared to the wild type (WT), both under Pi-starvation and Pi-sufficient conditions, and also showed a higher root biomass compared to WT in the Pi-starvation conditions. Seven days after the P-deficiency treatment, 85 distinct analytes were identified in the roots and 27 in the shoots between the Oe1 plant and WT, in which sophorose, sorbitol (sugars), hydroxybutyric acid (organic acids), and ornithine (amino acid) of *CmPht1;2* overexpressing chrysanthemum are specific responses to P-starvation.

## Introduction

As one of the major macronutrients, phosphorus (P) plays an important role in plant biochemical synthesis, energy transport, and signal transduction pathways (Wang et al., 2017). In addition, P is also involved in metabolism and the regulation of enzyme activity (Clarkson and Hanson, 1980). Although large amounts of phosphate-based fertilizer are applied in the agriculture system, most phosphorus in the soil becomes immobilized by precipitation or adsorption by soil minerals (Raghothama, 2000), resulting in a very low concentration of Pi in the soil solution available for plants (Vandamme et al., 2016). In this way, Pi-deficiency often limits plant growth and development (Raghothama and Karthikeyan, 2005).

PHOSPHATE TRANSPORTER1 (Pht1) family is plasma membrane-localized high-affinity Pi transporters and works on the uptake of inorganic phosphate (Pi) (Młodzińska and Zboińska, 2016). Nine Pi transporters in *Arabidopsis thaliana* were identified (Muchhal et al., 1996), and a number of homologs of PHT1 transporters have been isolated from other species (Rausch and Bucher, 2002). In general, Pht1 members consist of 12 transmembrane (TM) domains with two parallel parts of six hydrophobic TM fragments (Saier, 2000). The functions of some Pht1 members have been identified. For example, Pht1;1 and Pht1;4 contribute to Pi uptake despite of the P status (Shin et al., 2004). Pht1;5 acts on the mobilization of Pi from P source to sink organs (Nagarajan et al., 2011).

Chrysanthemum (*Chrysanthemum morifolium* Ramat.) is one of the most important ornamental species. P deficiency in soil is one of the limitations hampering the growth and ornamental value of cut chrysanthemum to a great extent, such as causing yellow spotted leaves and slow growth rate (Liu et al., 2013). In our previous study, we found a putative high-affinity phosphate transporter CmPT1 in chrysanthemum (Liu et al., 2014). In this study, we further characterized one of the Pht1 members, *CmPht1;2*, from a phosphorus-deficiency-tolerant chrysanthemum cultivar ‘Nannongyinshan’. The effects of its overexpression on the improvement of uptake of Pi and root dry weight of chrysanthemum was investigated. Moreover, the untargeted metabolic profiles were mined in *CmPht1;2* overexpressing chrysanthemum, to reveal information about plant biochemical biosynthesis, energy transfer reactions, and signal transduction events (Hernández et al., 2009; Bielecka et al., 2015; Ganie et al., 2015) under P starvation. This study provides the foundations for the improvement of phosphorus use efficiency in chrysanthemum.

## Materials and Methods

### Plant Materials and Growth Conditions

The phosphorus-deficiency-tolerant chrysanthemum cultivar ‘Nannongyinshan’ (Liu et al., 2014) was obtained from the Chrysanthemum Germplasm Resource Preserving Centre, Nanjing Agricultural University, China. The cuttings were rooted in aerated water without any nutrition in a greenhouse for 2 weeks. Then, they were transferred to a hydroponic solution consisting of a diluted (1:4) and (1:2) Hoagland’s solution for 3 days each (Bentonjones, 1982). Plants were maintained in the hydroponic solution with HP (300 μM, Pi) for 1 week, after which they were transferred to a hydroponic solution supplemented with either HP or –P (0 μM, Pi) for phosphate starvation treatment. The nutrient solution was renewed every 3 days. For *CmPht1;2* transcription profiles’ analysis, leaf, stem, and root tissues were harvested 7 days after the phosphate treatment, snap-frozen in liquid nitrogen, and kept at -80°C. Experiments included three replicates. Each replicate contained nine seedlings.

### Isolation and Sequence Analysis of *CmPht1;2*

The total RNA from the above sampled roots was extracted using Trizol reagent (Life Technologies) according to the manufacturer’s instructions. To amplify a full-length sequence of *CmPht1;2*, PCR primers (Full-F/R; **Supplementary Table S1**) were designed according to the transcriptome of *Chrysanthemum nankingense* (Ren et al., 2014). The full-length *CmPht1;2* cDNA sequence was transcribed using *Pfu* DNA polymerase (TaKaRa Ex Taq^®^). The open reading frame (ORF) of *CmPht1;2* was identified using the sequence analysis program (BioXM2.6). Sequences of multiple peptides were aligned using the DNAMAN software version 6, and phylogenetic analyses were performed using the MEGA v5.0 software.

### *CmPht1;2* Expression Patterns

The total RNA was isolated from the above sampled root, stem, and leaf tissues of plants grown under the HP (300 μM, Pi) or –P (0 μM, Pi) treatments for 7 days by Trizol; 500 ng⋅μL^-1^ RNA was used for cDNA synthesis. For quantification of *CmPht1;2*, real-time quantitative PCR (qPCR) assays were performed using the SYBR Green master mix (SYBR Premix Ex Taq^TM^ II, TaKaRa Bio) and the primer pair q*GSP*-F/-R (**Supplementary Table S1**) (Gao J. et al., 2016). The reference gene, *CmEF1α*, was amplified using the primers *EF1A*-F/-R (sequences given in **Supplementary Table S1**). Relative transcription levels were calculated by the 2^-ΔΔC_t_^ method (Kenneth and Livak, 2001).

### Construction of a GFP Fusion Vector and Intracellular Localization Analysis

The plasmid pENTR^TM^1A-*CmPht1;2* was confirmed by restriction enzyme *Dra* I and *Not* I digestion and DNA sequencing. pENTR^TM^1A-*CmPht1;2* was used to construct a C-terminus green fluorescent protein (GFP) fusion vector pMDC43-*CmPht1;2* by the LR reaction (as described in Gateway^®^ Technology with Clonase^®^ II). Plasmid DNA was bombarded into onion (*Allium cepa*) epidermal cells using a gene gun (PDS-1000; Bio-Rad, Hercules, CA, United States). The epidermal cells were incubated on Murashige and Skoog (MS) solid media plates in the dark for 16–20 h. The expression of GFP was monitored by confocal laser scanning microscopy at 488 nm (Zeiss, Germany) (Wan et al., 2008).

### Regeneration of *CmPht1;2* Overexpressing Chrysanthemum

To further analyze the function of *CmPht1;2*, the pMDC43-*CmPht1;2* vector was transformed into leaf disks of the phosphate deficiency sensitive cultivar ‘Jinba’ (Liu et al., 2014) via *Agrobacterium tumefaciens*-mediated transformation, using strain EHA105 (Höfgen and Willmitzer, 1988) as previously described (Li et al., 2017). The hygromycin (Hyg)-resistant plants were verified by the PCR analysis using the vector primer *Hyg*-F/-R (sequences given in **Supplementary Table S1**), and the overexpression of *CmPht1;2* was validated by qRT-PCR using primer pairs of *qGSP*-F/-R (sequences given in **Supplementary Table S1**).

### Phosphorus Uptake Assay of *CmPht1;2* Overexpressing Chrysanthemum

For phosphorus uptake velocity assay (^32^P uptake assay), transgenic lines and non-transformed WT plants were cultured at HP (300 μM Pi) conditions for 1 week, followed by a 7-day hydroponic culture in HP (300 μM Pi) and LP (15 μM Pi) solutions, respectively. Subsequently, plants were incubated in 100 mL hydroponic culture with HP (300 μM Pi) containing 1.2 μCi or LP (15 μM Pi) containing 0.06 μCi of H_3_^32^PO_4_ for 3 and 6 h. Then, whole seedlings were rinsed thoroughly in sterile ddH_2_O, dried at 80°C, and fine grounded. A scintillation solution was then added to the ground samples. The radioactivity of the mixture was detected with a Beckman LS6500 scintillation counter.

Transgenic lines and non-transformed wild-type (WT) plants were cultured at HP conditions for 1 week, followed by hydroponic culture in either P-sufficient (300 μM Pi; HP) or P-deficiency (15 μM Pi; LP) conditions for 7 days. The roots and shoots were harvested for the total phosphorus or inorganic phosphate concentration assay. The total plant phosphorus concentration was measured by a proton spectrometer from a ∼0.1 g dry sample as previously described (Chen et al., 2010). The inorganic phosphate concentration was calculated (Zhou et al., 2008). Experiments included three replicates, and each replicate contained three seedlings.

### Morphological Characteristics of the *CmPht1;2* Overexpressing Chrysanthemum

Seedlings were subjected to HP or LP treatment as mentioned above. After 1-week HP or LP treatment, the root length and plant height of the seedlings were measured. The biomasses of roots and shoots were determined by dry weight. The root architecture was measured using a root scanner (Epson Color Image Scanner LA1600+). The experiments included three replicates, and each replicate included three seedlings.

### Metabolomics Sample Preparation

*CmPht1;2-*overexpressing plants from the Oe1 line and WT plants were grown in a hydroponic solution under HP conditions for 1 week and then transferred to P-starvation (15 μM Pi; LP) medium for 7 days. The roots and leaves were harvested at 0, 2, and 7 days after the P-deficiency treatment. Six plants were included for each time point.

A root or shoot sample of 60 mg (dry weight) was transferred to 360 μL cold methanol, which was ground and ultrasonicated at the ambient temperature for 30 min immediately; 200 μL of chloroform was added, samples were vortexed, followed by adding 400 μL ddH_2_O, and subsequently centrifuged at 12,000 rpm for 10 min at 4°C. The mean value of all samples were pooled to act as a quality control (QC). An aliquot of the 500 μL supernatant was taken as previously described (Peng et al., 2015) and incubated at 37°C for 90 min.

The analysis of metabolites was performed on an Agilent 7890B gas chromatography system coupled to an Agilent 5977A MSD system (Agilent Technologies Inc., Santa Clara, CA, United States). Derivatives were separated as described previously (Zhou et al., 2012). Helium (>99.999%) was used as the carrier gas at a constant flow rate of 1 mL/min. The injector temperature was maintained at 260°C. The injection volume was 1 μL by splitmode with a split ratio of 4:1. The initial oven temperature was 60°C, ramped to 125, 210, 270, and 305°C, and finally held at 305°C. The temperatures of the MS quadrupole and the ion source (electron impact) were set to 150 and 230°C, respectively. The mass data were obtained from a full-scan mode (m/z 50–500).

### Data Preprocessing and Statistical Analysis of GC-MS

The differential metabolites were identified by the statistically significant threshold of variable influence on projection (VIP) values (VIP > 1, *P* < 0.05). OPLS-DA (orthogonal partial least-squares-discriminant analysis) was performed to visualize the metabolic difference of roots/shoots among the Oe plants and WT plants in response to LP stress after mean centering and unit variance scaling. Those differential metabolites between transgene lines and WT plants in response to LP were identified with a threshold (upregulate, FC > 1.2, and downregulate, FC < 0.8) as described previously (Lan et al., 2011). The relevant metabolic pathways were identified from the database of KEGG, and the significant changes were based on *P* < 0.01.

### Statistical Analysis

All the significance discriminate analyses were carried out using the SPSS software (IBM SPSS Statistics Version 20), and the phenotypes of the Oe lines and WT were analyzed using Student’s *t*-test at 5% level of probability. The figures were spliced using Photoshop software.

## Results

### Cloning and Sequence Analysis of *CmPht1;2*

**FIGURE 1:**
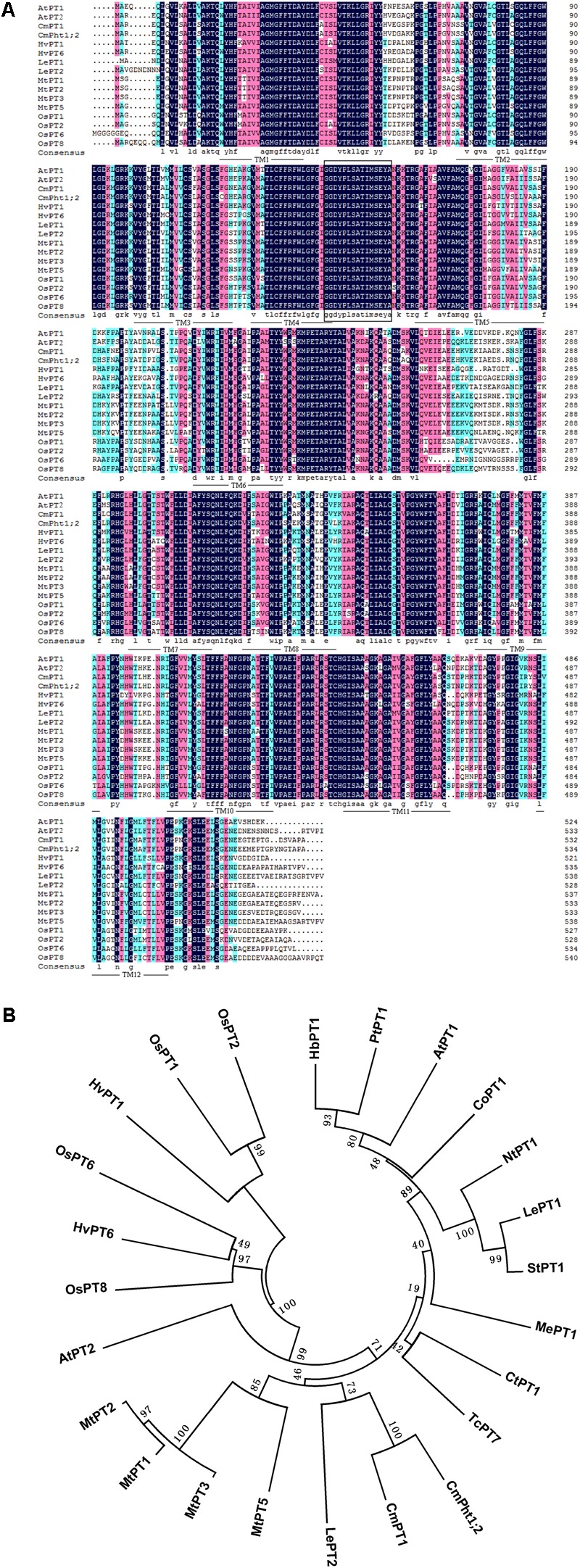
Alignment and phylogenetic relationships of the peptide sequences of high-affinity Pi transporters. **(A)** Alignment of amino acid sequences of PT1 members. Identical peptides are highlighted in black and conserved substitutions in pink. Putative CmPht1;2 transmembrane domains are underlined. The Pht1 signature sequence is boxed. **(B)** Phylogenetic relationships of the peptide sequences of PT1s. AtPT1 (AED94948) and AtPT2 (AAC79607) from *Arabidopsis thaliana*; HvPT1 (AAO32938) and HvPT6 (AAN37901) from *Hordeum vulgare*; MtPT1 (AAB81346), MtPT2 (AAB81347), MtPT3(ABM69110), and MtPT5 (ABM69111) from *Medicago truncatula*; LePT1 (AAB82146) and LePT2 (AAB82147) from *Lycopersicon esculentum*; OsPT1 (Q8H6H4), OsPT2 (Q8GSD9), OsPT6 (Q8H6H0), and OsPT8 (Q8H6G8) from *Oryza sativa*; CtPT1 (AFY06657) from *Citrus trifoliata*; TcPT1 (EOY06027) from *Theobroma cacao*; CoPT1 (AFU07481) from *Camellia oleifera*; NtPT1 (AAF74025) from *Nicotiana tabacum*; StPT1 (AAD38859) from *Solanum tuberosum*; MePT1 (ADA83723) from *Manihot esculenta*; HbPT1 (ADL27918) from *Hevea brasiliensis*; PtPT1 (XP_002306844) from *Populus trichocarpa*; and CmPT1 (AGK29560) from *Chrysanthemum morifolium*.

The full length of *CmPht1;2* was a 2,105 bp sequence, containing a 1,605 bp ORF which encodes a 534 amino acid polypeptide (**Figure 1A**). The calculated molecular mass and pI of the CmPht1;2 protein were 58.41 kDa and 8.12, respectively. The peptide was predicted to have 12 transmembrane domains (TMs), including a hydrophilic loop in the middle of the domains and a Pht1 signature sequence GGDYPLSATIxSE between TM4 and TM5 (**Figure 1A**). The identity level of the CmPht1;2 amino acid sequence with Pi transporters from other plant species ranged from 68.0% (*Hordeum vulgare* HvPT1, AAO32938) to 79.2% (*Lycopersicon esculentum* LePT1, AF022874). The sequence identity between chrysanthemum gene CmPht1;2 and CmPT1 (AGK29560) (Liu et al., 2014) is 90.8% (**Figure 1B**).

### *CmPht1;2* Transcription Induced by Pi-Deficient Conditions

**FIGURE 2:**
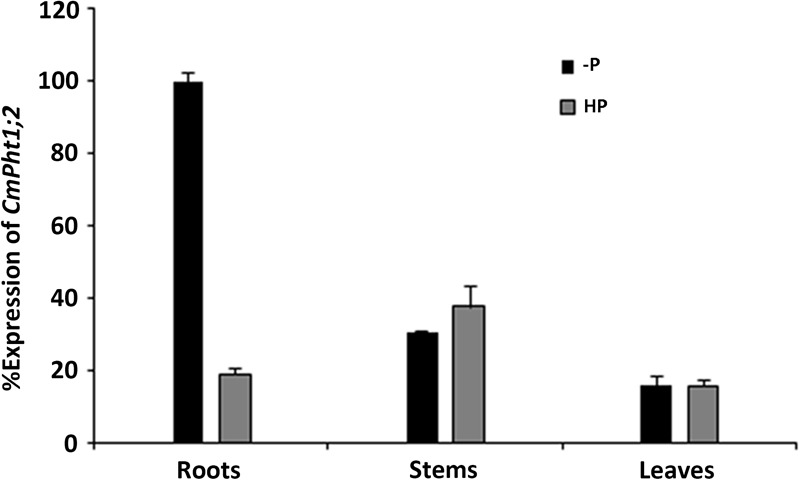
Relative transcript abundance in the roots, stems, and leaves under HP (300 μM Pi) and -P (0 μM Pi).

*CmPht1;2* transcriptions were present in roots, stems, and leaves, and were highest in the stem under Pi-sufficient (300 μM Pi; HP) conditions. However, it was strongly induced (5.3 times) in Pi-deficient (0 μM Pi; -P) conditions in the roots, while the induced expression was not obvious in the stems and leaves of chrysanthemum exposed to -P conditions (**Figure 2**).

### Plasma Membrane Localization of CmPht1;2

**FIGURE 3:**
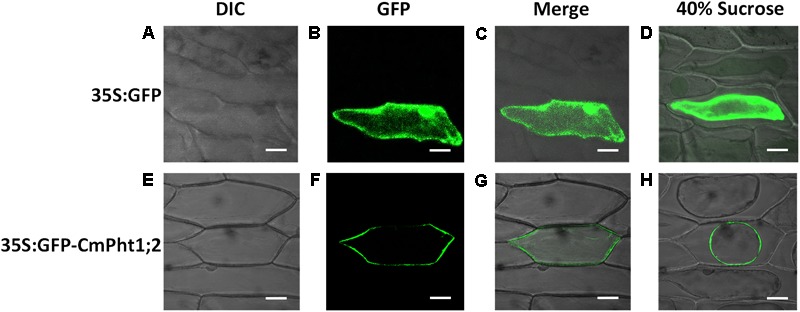
Subcellular localization analysis of CmPht1;2. **(A–D)** Onion epidermal cells transformed with 35S:GFP. **(E–H)** Onion epidermal cells transformed with 35S:GFP-CmPht1;2. **(A,E)** Bright field microscopy images to display morphology. **(B,F)** Dark field images for detection of GFP fluorescence. **(C,G)** Superimposed light and dark field images. **(D,H)** Plasmolyzed onion epidermal cells. Bar: 50 μm.

To investigate the intracellular localization of the CmPht1;2 transporter, we analyzed a GFP-CmPht1;2 construct driven by the 35S promoter of the cauliflower mosaicvirus in onion epidermal cells. The GFP signals presented throughout the analyzed cells, including the nucleus, cytoplasm, and plasma membrane of the onion epidermal cell transformed with the pMDC43:35S-GFP empty vector (as control). The fluorescence signal was mainly observed on the plasma membrane of onion epidermal cells transformed with 35S:GFP-CmPht1;2, and this observation was confirmed by observing plasmolyzed cells (**Figure 3**).

### Overexpression of *CmPht1;2* in Chrysanthemum Facilitated P Uptake

**FIGURE 4:**
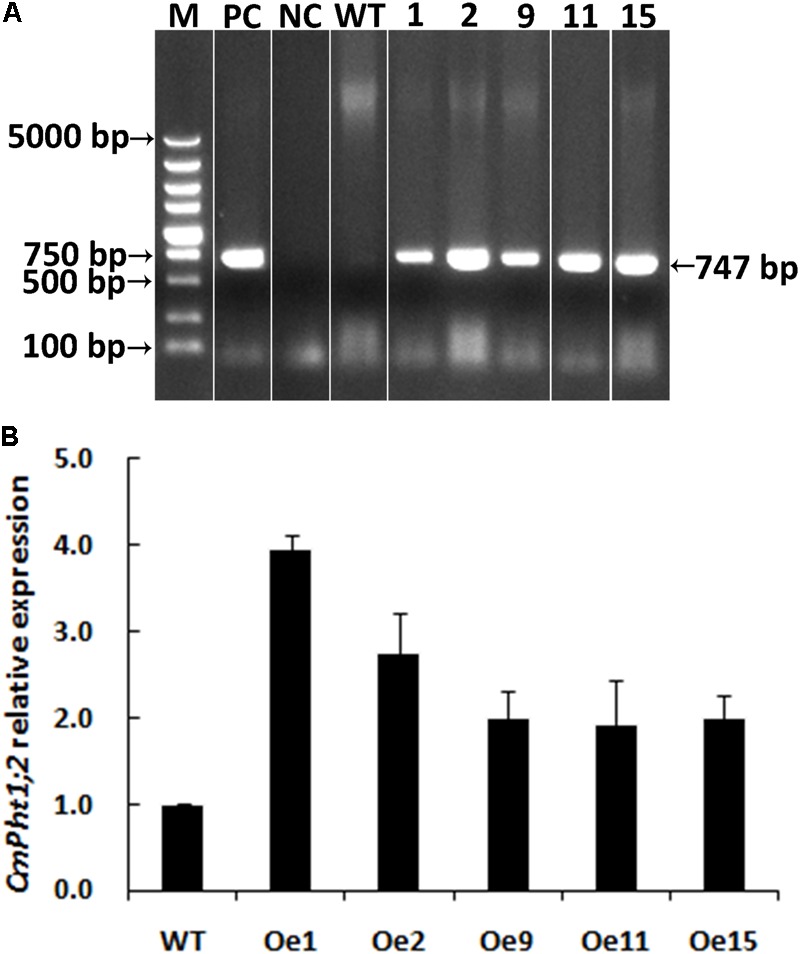
Validation of transgenic plants. **(A)** PCR analysis of genomic DNA extracted from hygromycin-resistant regenerants. **(B)** Relative *CmPht1;2* transcript abundance in wild-type (WT) and transgenic plants. The figure was spliced and grouped using Photoshop software.

A genetic transformation system for ‘Nannongyinshan’ is not available. Therefore, to identify the function of *CmPht1;2, CmPht1;2* was introduced into the low Pi (15 μM Pi; LP) sensitive cultivar ‘Jinba’ by agrobacterium-mediated leaf disk transformation. Five putative transgenic plants were identified by the PCR analysis using *Hyg* primers (**Figure 4A** and **Supplementary Figure S1**). Five overexpression lines were validated by qRT-PCR, of which Oe1 and Oe2 lines displayed the highest *CmPht1;2* abundance (3.96- and 2.74-folds compared to that of WT), and were thus selected for further study (**Figure 4B**).

**FIGURE 5:**
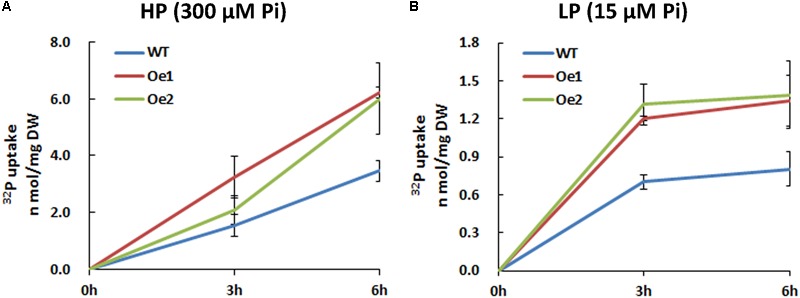
^32^Pi uptake velocity in *CmPht1;2*-overexpressing lines. Pi uptake activity of WT and transgenic plants (Oe1 and Oe2) under HP (300 μM Pi) **(A)** and LP (15 μM Pi) **(B)** conditions. Error bars represent SD (*n* = 3). Three plants per line were included.

The Oe lines and WT plants were subjected to either HP or LP solutions for the functional analysis of *CmPht1;2*. Compared with WT plants, the Pi uptake of the overexpressing lines increased significantly under both HP and LP status (**Figure 5**).

**FIGURE 6:**
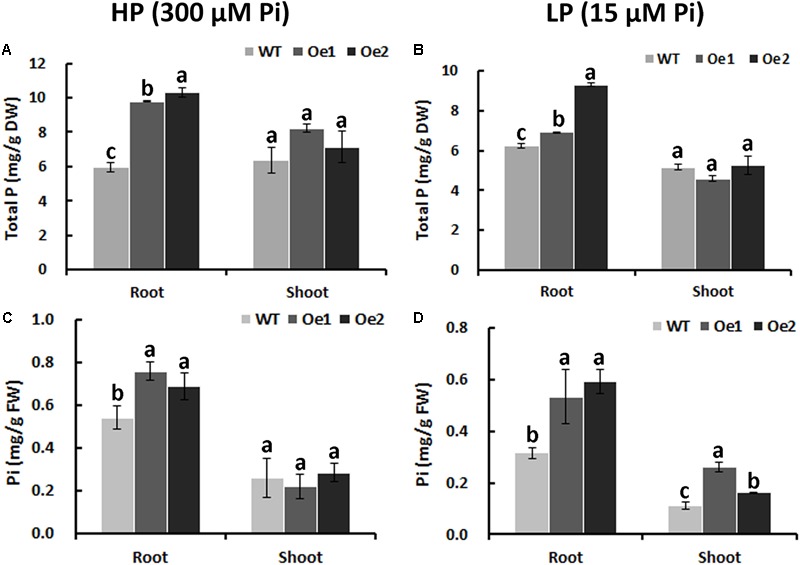
Pi uptake in *CmPht1;2*-overexpressing lines. Total P contents in roots and shoots were measured in WT and transgenic plants (Oe1 and Oe2) under HP (300 μM Pi) **(A)** or LP (15 μM Pi) **(B)**. Error bars represent SD (*n* = 3). DW, dry weight. Pi contents in roots and shoots under HP (300 μM Pi) **(C)** or LP (15 μM Pi) **(D)**, respectively. Error bars represent SD (*n* = 3). FW, fresh weight. Three plants per line were included.

Under HP status, the total P content in the roots of Oe1 and Oe2 plants increased by 64.7 and 73.7%, respectively, compared with WT plants, while no obvious difference in P concentrations of the shoots between Oe lines and WT plants was observed (**Figure 6A**). Under LP conditions, the total P concentrations in Oe lines were improved by 11.1 and 49.4%, respectively, in the roots compared with the WT plants, but the concentration of P in the shoots of Oe lines was comparable to that observed in the WT (**Figure 6B**). Inorganic phosphorus concentration in the roots of Oe1 and Oe2 increased by 40.0 and 27.0%, respectively, while those in the shoots had no obvious difference compared with the WT plants under HP conditions (**Figure 6C**). Under LP conditions, inorganic phosphorus concentrations in the roots of Oe1 and Oe2 plants were improved by 68.5 and 87.5%, respectively, and in the shoots, they increased by 133.8 and 45.4% compared with WT (**Figure 6D**).

### Overexpression of *CmPht1;2* Increased Shoot Height, Root Length, Root Biomass, and Number of Root Tips and Forks of Chrysanthemum

**FIGURE 7:**
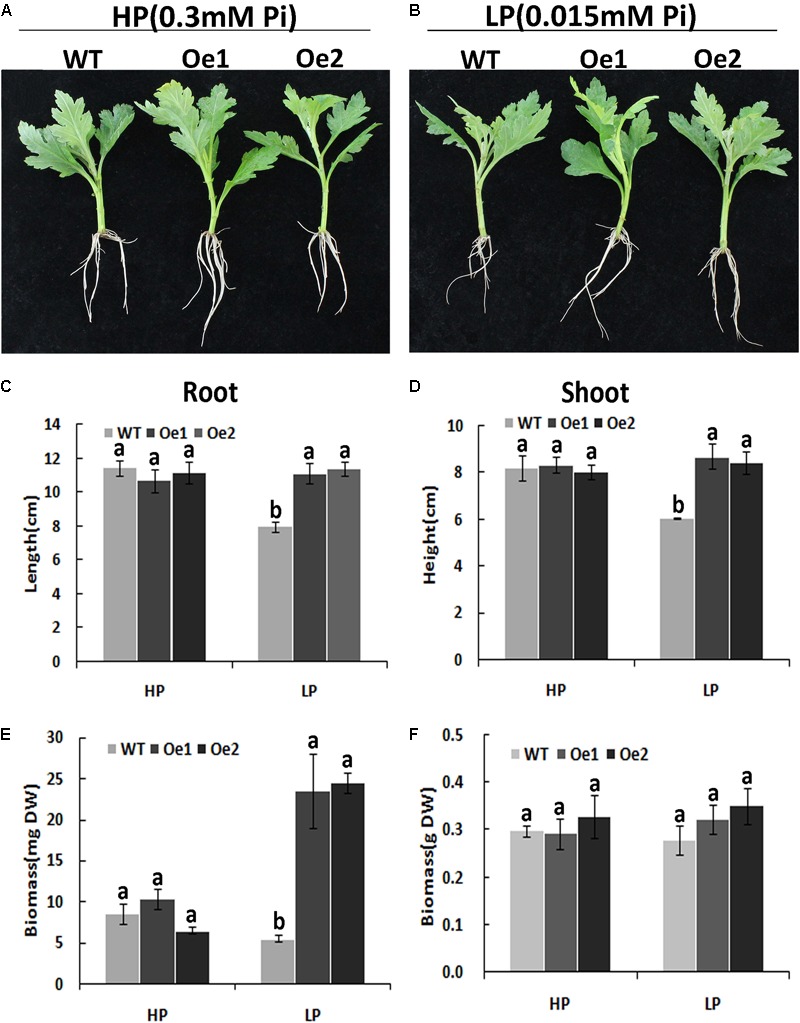
Growth performance of the WT and *CmPht1;2*-Oe lines at different Pi levels in a hydroponics experiment. Growth performance of WT and *CmPht1;2-*overexpressing plants (Oe1 and Oe2) at HP (300 μM Pi) **(A)** and LP (15 μM Pi) **(B)** conditions. Roots **(C)** and shoots **(D)** height of WT and transgenic plants under HP and LP conditions. Biomass measurements of roots **(E)** and shoots **(F)** were obtained from WT and transgenic plants grown in nutrient solution to which 300 μM Pi (HP) or 15 μM Pi (LP) were added. DW, dry weight. Three plants per line were included.

**FIGURE 8:**
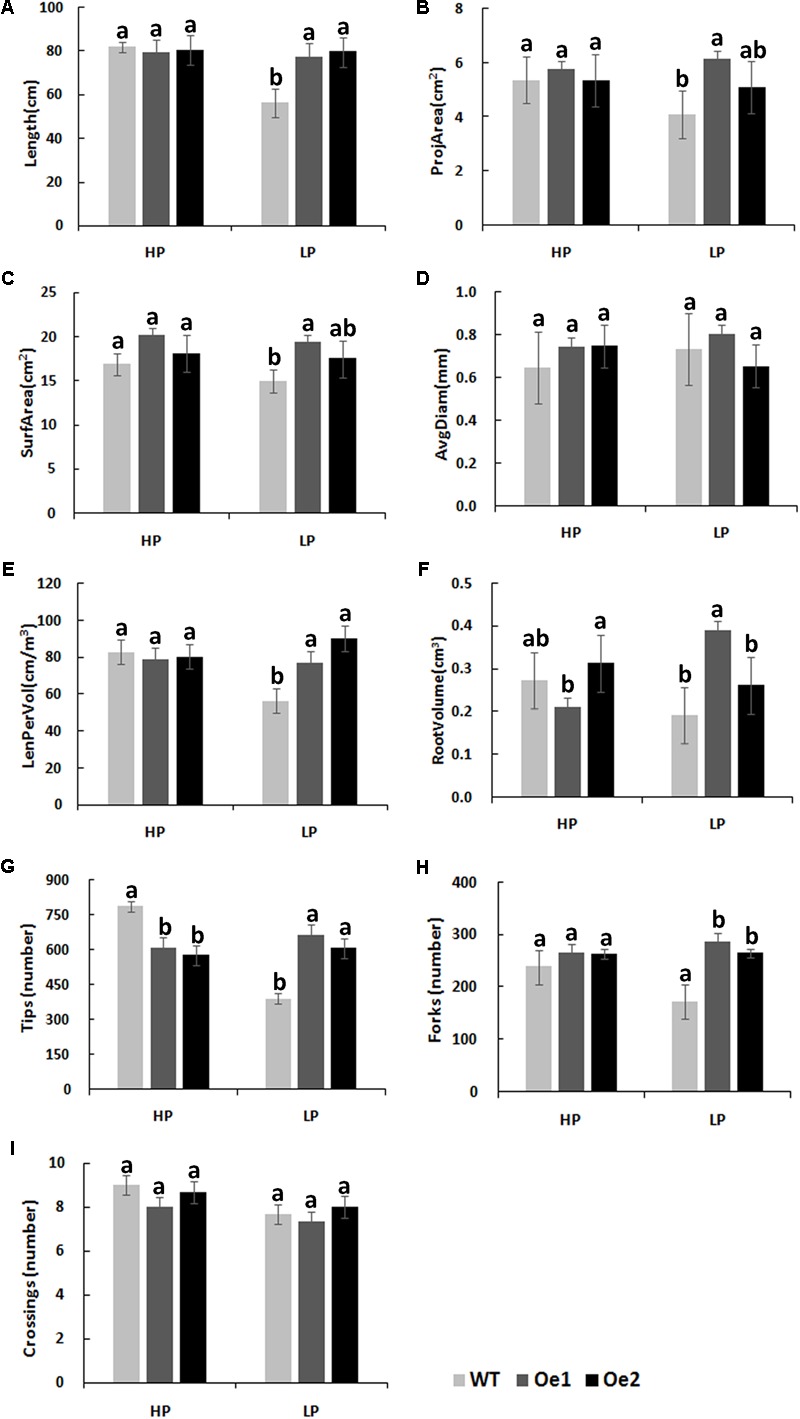
Root architecture of *CmPht1;2* overexpressing plants under HP or LP conditions enhanced. **(A)** Total lengths (Length). **(B)** Projected area (ProjArea). **(C)** Superficial area (SurfArea). **(D)** Average projected area (AvgDiam). **(E)** Length per volume (LenPerVol). **(F)** Root volume. **(G)** Numbers of root tips (Tips). **(H)** Numbers of root forks (Forks). **(I)** The branch roots (Crossings). Error bars represent SD (*n* = 3). Three plants per line were included.

Under HP conditions, no significant differences in plant height, root length, and biomass of roots and shoots were observed between Oe lines and WT (**Figure 7**). In contrast, under LP conditions, the root length of Oe1 and Oe2 lines increased by 39.6 and 43.0%, respectively, and the plant height was enhanced by 42.7 and 38.8%, respectively (**Figures 7C,D**). The dry biomasses of roots of Oe1 and Oe2 were 4.27 and 4.45 times more than that of WT plants, and the biomasses of shoots were 1.15- and 1.26-folds more than that of WT under LP stress (**Figures 7E,F**). The root scanner analysis showed that root morphogenesis was similar in Oe lines and WT lines under HP conditions (**Figure 8**), except that the numbers of root tips slightly decreased by 29.3 and 36.7% in Oe1 and Oe2, respectively (**Figure 8G**). In contrast, under the LP conditions, the total lengths of roots of Oe1 and Oe2 increased by 37.2 and 41.1%, respectively, in comparison to WT (**Figure 8A**). Similarly, the projected area (ProjArea) of the two transgenic plants was increased by 50.7 and 24.5%, respectively, compared to WT (**Figure 8B**); the surface area (SurfArea) by 29.3 and 16.8% (**Figure 8C**), the length per volume (LenPerVol) by 37.2 and 60.0% (**Figure 8E**), the root volume by 100.3 and 37.0% (**Figure 8F**), the number of root tips by 69.6 and 55.8% (**Figure 8G**), and the number of root forks by 66.6 and 53.9% (**Figure 8H**). Instead, the average projected area (AvgDiam) and branch roots (Crossings) showed no obvious difference between Oe lines and WT (**Figures 8D,I**).

### Differential Metabolites and Metabolic Pathways in *CmPht1;2* Overexpressing Plants in Response to Pi Deficiency

**FIGURE 9:**
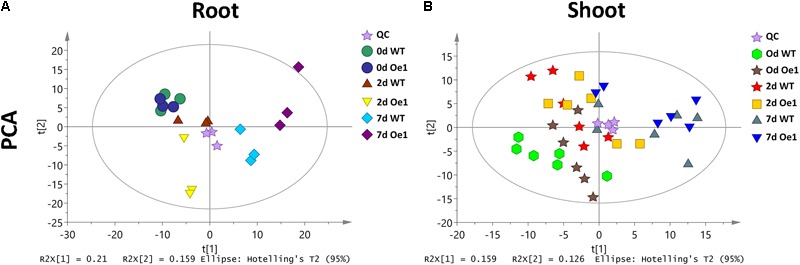
Principle component analysis (PCA) score plot of WT and *CmPht1;2* Oe1 line. PCA score plot of roots **(A)** and shoots **(B)** of WT and *CmPht1;2* Oe1 line under 0, 2, and 7 days Pi stress, respectively.

Non-targeted metabolite profiles were investigated in the roots and shoots of *CmPht1;2* overexpressing plants (Oe1 which have representative phenotypes) and WT subjected to LP treatment for 0, 2, and 7 days. The principle component analysis (PCA) showed that the metabolite profiles in the roots and shoots of Oe1 plants differed from WT plants in response to LP stress (**Figure 9**). In the roots, there were 19 different metabolites between Oe1 and WT plants at day 0, 40 different metabolites at day 2, and 85 different metabolites at day 7 under the LP conditions (**Supplementary Table S2**). In the shoots, differential metabolites between Oe1 and WT plants at day 0 were 27, day 2 (43), and day 7 (27) under LP conditions (**Supplementary Table S3**).

There was an increase in amino acids including *N*-methyl-L-glutamic acid and tryptophan, organic acids of lactobionic acid, and dehydroshikimic acid in the roots of Oe1 compared with WT at day 0. Compared with WT, sugars such as galactinol, isopropyl-beta-D-thiogalactopyranoside, and sorbitol were upregulated in the roots of Oe1 plants at day 2 under LP conditions. While at day 7 under LP stress, the main compounds in the roots of the Oe1 plants that increased were the amino acid of *N*-alpha-acetyl-L-ornithine, phenol of 4-vinylphenol dimer, pigment of alizarin, and organic acid of 3-hydroxybutyric acid compared with that of WT, whereas aminooxyacetic acid and *cis*-gondoic acid in the roots of Oe1 plants were less abundant than those in WT plants at day 0; 3-phenyllactic acid, butyraldehyde, cysteinylglycine, and nicotianamine in the roots of Oe1 plants showed a decline compared with those in WT plants at day 2. In addition, galactinol, glucoheptonic acid, nicotianamine, nicotinamide, salicylic acid, and sucrose decreased in the roots of Oe1 plants at 7-day post-treatment compared to the WT plants (**Supplementary Table S2**).

Compounds upregulated in the shoots of Oe1 plants relative to the WT plants included energy metabolites (glucose-6-phosphate and *O*-succinyl homoserine), amino acids (*N*-acetyltryptophan and L-glutamic acid), and organic acids (3-hydroxypropionic acid and 4-acetylbutyric acid) at 0 day, a slight increase in sugars (fructose, sophorose, and tagatose) at 2 days under LP stress, and increases in phenols (4-hydroxy-3-methoxybenzyl alcohol), nucleotide (adenosine), and amino acid (lysine) at 7 days after the LP treatment. However, amino acids (3-hydroxy-L-proline) and organic acids (3-hydroxybutyric acid) showed a decrease in the shoots of Oe1 plants in comparison to WT at 0 day. Most of the nucleotides and their degraded products (5,6-dihydrouracil, cytidine-monophosphate, and flavin adenine), in addition to amino acids (isoleucine) and organic acids (allylmalonic acid), decreased drastically in the shoots of Oe plants in comparison to WT plants following 2-day LP stress. Additionally, sterols (20-α-hydroxycholesterol and 4-cholesten-3-ketone) and organic acids (3-hydroxy-3-methylglutaric acid) also decreased in the 7-day-P-starved shoots of Oe1 plants compared to WT plants (**Supplementary Table S3**).

Significantly changed pathways in the roots of Oe1 plants vs. WT mainly consisted of hormone biosynthesis, sugar metabolism, alkaloid biosynthesis, and especially energy and amino acid metabolism pathways (**Supplementary Tables S4–S10**).

**FIGURE 10:**
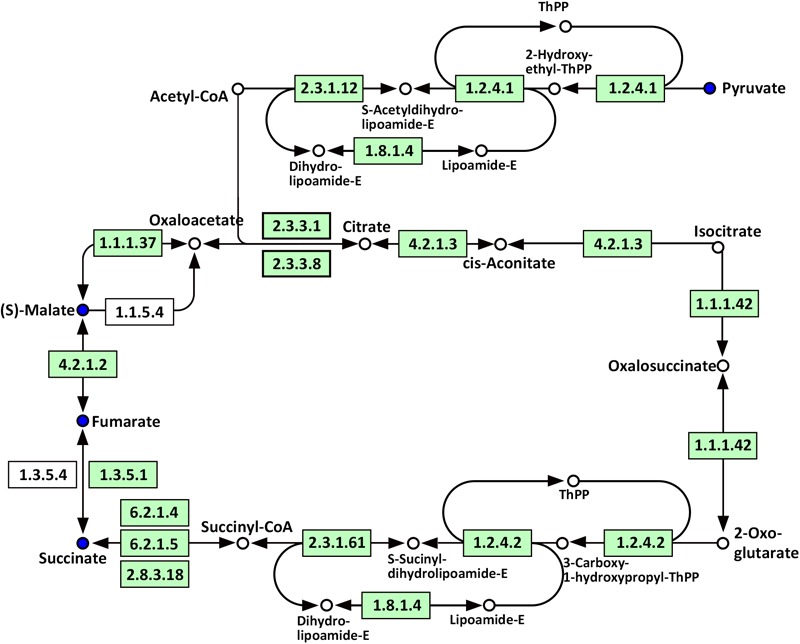
Mapping of the TCA cycle pathways involved in *CmPht1;2* Oe1 in response to LP compared with WT based on KEGG. Blue circles indicate metabolites that were downregulated in *CmPht1;2* Oe1 compared with WT. Boxes indicate the enzymes involved in this pathway.

Compared with WT, significantly altered energy metabolism pathways in the roots of Oe1 plants after the LP treatment (especially at day 7) included the citrate cycle (TCA cycle), glyoxylate and dicarboxylate metabolism, carbon fixation in photosynthetic organisms, galactose metabolism, and glycolysis/gluconeogenesis (**Supplementary Table S4**). It is noteworthy that succinate, fumarate, malate, and pyruvate were all downregulated at 7-day LP stress in the Oe1 roots compared with WT (**Figure 10**).

**FIGURE 11:**
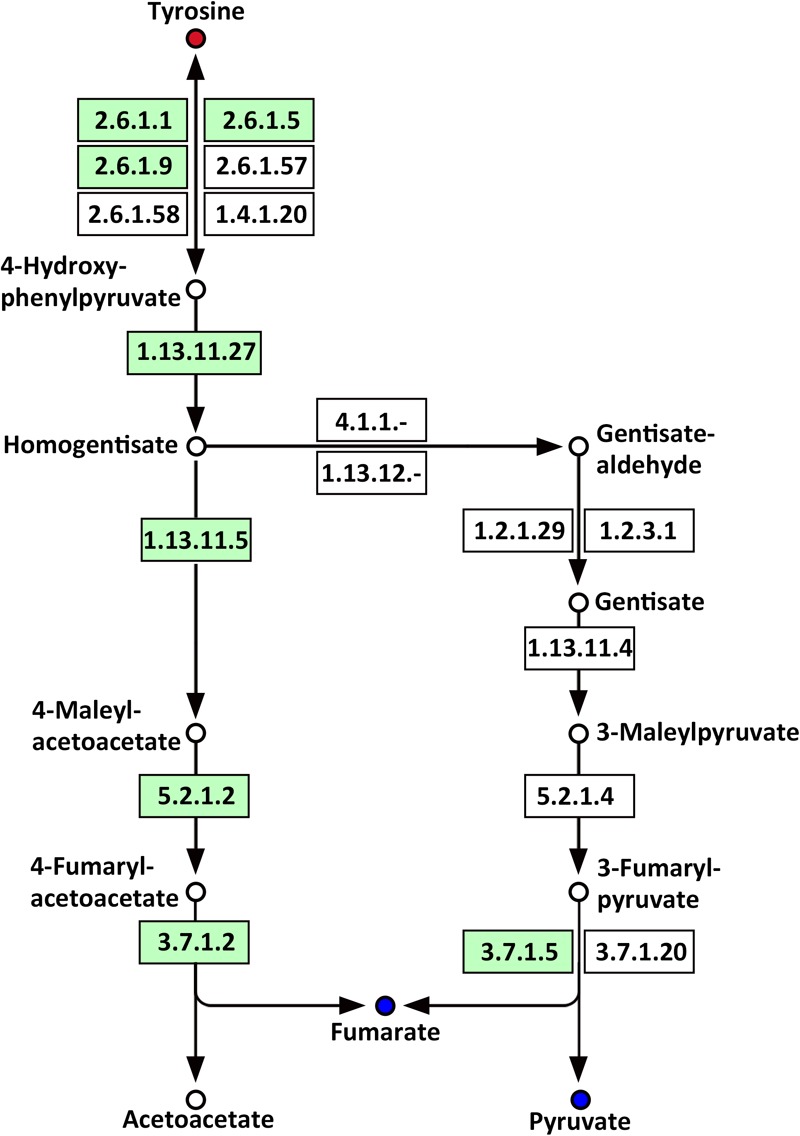
Mapping of the tyrosine metabolism pathways involved in *CmPht1;2* Oe1 response to LP compared with WT based on KEGG. Blue circles indicate metabolites that were downregulated in the *CmPht1;2* Oe1 compared with WT. Red circles indicate metabolites that were upregulated in*CmPht1;2* Oe1 compared with WT. Boxes indicate the enzymes involved in this pathway.

Several amino acids related to metabolic pathways showed a clear change in response to LP treatment in the roots of Oe1 in comparison with WT (**Supplementary Table S4**). For example, tyrosine (a precursor of the tyrosine metabolism pathway) was present in a higher abundance in the roots of Oe1 plants compared to those found in WT at 7 days of LP stress (**Supplementary Table S2**). Other compounds such as fumarate and pyruvate, which act as products of tyrosine, decreased in most metabolic pathways of Oe1 roots compared to WT under LP conditions (**Figure 11**).

## Discussion

### CmPht1;2 Is an Inducible Pi Transporter Located at the Plasma Membrane

Phosphorus in the plant, an essential nutrient, plays an important role both at the vegetative and reproductive stages (Shi et al., 2014). Various Pi transporters including the Pht1 family are identified to function on the Pi uptake and translocation throughout the plants (Nagarajan et al., 2011). We previously isolated a putative chrysanthemum Pi transporter CmPT1 which mediated Pi uptake in chrysanthemum (Liu et al., 2014). In this study, *CmPht1;2* was found to have the typical structure and signature sequence of Pht1 family members (**Figure 1A**), suggesting that *CmPht1;2* is also a member of the chrysanthemum Pht1 family. In addition, the predicted transmembrane domains based on the amino acid sequence, and localization at the plasma membrane in transfected onion cells, suggested that CmPht1;2 is a transmembrane protein (**Figure 3**), which is in consistent with previous studies that the Pht1 family consisted of a group of Pi transporters located in the plasma membrane (Nussaume et al., 2011). The majority of *Arabidopsis* PHT1 transporters are expressed exclusively in roots, where they are induced by P-starvation (Ai et al., 2009), consistent with their major function of Pi uptake from the rhizosphere (Teng et al., 2017). In the present study, the *CmPht1;2* transcript abundance was strongly induced in the roots under phosphate-deficient conditions (**Figure 2**), suggesting that CmPht1;2 might be associated with Pi uptake under LP stress.

### CmPht1;2 *in Planta* Functions in Pi Uptake and Enhances Root Growth

The membrane transporters play key roles in plant nutrient uptake (Saia et al., 2015). In the present study, the Pi uptake rate was improved under both HP and LP conditions (**Figure 5**). In addition, the total content of phosphate in the root of Oe lines was notable higher than WT (**Figure 6**), indicating that CmPht1;2 is essential for Pi acquisition from rhizosphere, which is in line with the observation that double mutants of *pht1;1* and *pht1;4* or single mutant significantly decreased Pi uptake (Misson et al., 2004; Shin et al., 2004). Here, the growth of WT chrysanthemum was arrested under Pi-deficient conditions, similar to previous observations in rice (Jia et al., 2011), whereas the *CmPht1;2* overexpression could alleviate the arrest (**Figure 7**). Similarly, the growth of tobacco cells overexpressing high-affinity phosphate transporter PHT1 was increased under the phosphate-starvation conditions (Mitsukawa et al., 1997). The overexpression of the *CmPht1;2* directly or indirectly caused Pi-dependent root architecture alterations with enhanced root elongation and proliferated lateral root growth and increased root area and root tips under LP condition (**Figure 8**). These changes enabled exploration of soil Pi with an improvement in the absorptive surface area of roots (Nagarajan et al., 2011). It is noteworthy that the parallel increases of PT transcript and protein levels were observed in tomato, indicating the transcriptional and translational regulation of the phosphate transporter genes (Muchhal and Raghothama, 1999). In the present study, the phenotype of Oe1 and Oe2 is partly inconsistent with the transcripts levels of *CmPht1;2*, which might be due to the unknown level of protein expression and the positional effect of integration of *CmPht1;2* into genome or other unknown mechanisms.

### Metabolic Profiles Are Altered in *CmPht1;2* Overexpression Line

Metabolomics provides tools to identify metabolic processes and analyze the physiological adaption of plants to different nutrient availabilities (Hirai and Saito, 2004). For example, untargeted metabolomic profiling of plants under sulfate-limited conditions and resupply provided whole metabolome responses to sulfur nutrition in Arabidopsis (Bielecka et al., 2015). In the present study, Oe1 plants showed a number of different metabolites compared with WT plants under LP treatment. The identified differential metabolites were mostly primary metabolites including amino acids, nucleotides, sugar, energy metabolism compounds, and organic acids (**Supplementary Tables S2, S3**). These metabolites belong to different pathways, such as energy metabolism and the amino acid metabolism/biosynthesis pathways (**Figures 10, 11**), indicating that the overexpression of *CmPht1;2* could affect the metabolic adaption of chrysanthemum to LP stress. Notably, a number of changes in the metabolism profiles were quite different from previous descriptions (Hernández et al., 2007; Hernández et al., 2009; Ganie et al., 2015). Where stress-related metabolites such as polyols accumulated in the P-deficient root of common bean (Hernández et al., 2007), glycerolipid metabolism and phenylalanine pathway have been identified in common bean under P stravation (Hernández et al., 2009). A sharp increase in asparagine, serine, and glycine was observed in both shoots and roots of maize under low P conditions (Ganie et al., 2015). To our knowledge, changes in sophorose, sorbitol (sugars), hydroxybutyric acid (organic acids), and ornithine (amino acid) are specific responses of *CmPht1;2* overexpressing chrysanthemum to LP conditions.

Phosphorus is the classical glycolysis-dependent cosubstrate (Plaxton, 2004) under LP conditions; higher availability of phosphorus in Oe1 plants may facilitate glycolysis in roots which is evidenced by the downregulation of disaccharides (sucrose) (**Supplementary Table S2**). Moreover, the upregulation of monosaccharides including galactinol, thiogalactopyranoside, and sorbitol implied that the roots of Oe1 plants might accumulate more carbohydrate for the growth (**Supplementary Table S2**). Sorbitol is a production of photosynthesis and an important translocated form of carbon, and is closely related to the growth and development of plants (Loescher et al., 1982). As a downstream pathway of glycolysis (Fernie et al., 2004), the contents of organic acids such as succinate, fumarate, malate, and pyruvate of the TCA cycle were less abundant in the roots of Oe1 plants than WT (**Figure 10**). These decreased organic acids in Oe1 plants under LP conditions could be the result of an enhanced secretion of organic acids into the rhizosphere, which in turn facilitated the Pi mobility in Oe1 plants’ rhizosphere (Plaxton, 2004; Yao et al., 2011). Amino acids such as glutamic acid, tryptophan, and ornithine were increased in the root of Oe1 plant vs. WT (**Supplementary Table S2**), which might be a consequence of the increased amino acids’ synthesis or repressed amino acids’ degradation. Similar responses to P starvation have been reported in maize (Ganie et al., 2015), suggesting a conserved responses of those amino acids of plants to Pi starvation across different species. Glutamic acid and ornithine were involved in the supply of nitrogen for the growth of roots (Walker et al., 1984; Wright et al., 1995), and tryptophan is a precursor for auxin biosynthesis (Gao Y. et al., 2016). The aromatic amino acids’ tyrosine which is a promoter of root length in plants (Bertin et al., 2007) was increased in Oe1 plants compared to WT under LP conditions (**Figure 11**). Taken together, the increases in glutamic acid, tryptophan, ornithine, and tyrosine might contribute to the enhanced root architecture in the roots of Oe1 than WT. Other compounds like nicotianamine (a chelator of metals) were increased as well (**Supplementary Table S2**), and it acts on the acquisition of iron in plants (Takahashi et al., 2003). If the Oe1 plant might possibly uptake more metals for the growth, additional evidence is needed before we could make a conclusion.

Twenty-seven compounds still had a sharp difference in shoots between Oe1 and WT plants under LP treatment (**Supplementary Table S3**). For example, fructose had a slight accumulation in the shoots of the Oe1 plant compared with WT, which possibly came from the degradation of phosphorylated polysaccharides, and consequently released more Pi (Morcuende et al., 2007). Similarly, most of the nucleotides, as organic P, significantly declined in the Oe plants vs. WT as well (**Supplementary Table S3**); similar changes have been found in maize (Ganie et al., 2015). Though the total P content had not obviously changed in the shoots between Oe1 and WT plants, the Pi concentration in the shoots of Oe1 plant increased (**Figure 6**). We suggested that the overexpression of *CmPht1;2* might directly or indirectly enhance the Pi homeostasis in shoots rather than facilitating a transport of Pi from the root to the shoot (Wu et al., 2013).

## Conclusion

In this study, we have identified the Pht1 family member *CmPht1;2*, whose expression is induced in the roots by P starvation. *CmPht1;2*-overexpressed chrysanthemum showed enhanced phosphorus uptake, suggesting that *CmPht1;2* may play a significant role in phosphate acquisition and root architecture reestablishment under LP. Metabolic profiles inferred that P participates in the regulation of amino acids and energy metabolism in chrysanthemum. The present study provides the basis for further studies on the Pi uptake modulation in chrysanthemum.

## Author Contributions

SC, FC, JJ, HW, and AS designed this study. CL did the main work of experimentation. All authors carried out the field experiments. YZ assisted deal with data of experiments. CL wrote the manuscript under the supervision of SC. JS and GS made a modification of the manuscript. All authors read and approved this manuscript.

## Conflict of Interest Statement

The authors declare that the research was conducted in the absence of any commercial or financial relationships that could be construed as a potential conflict of interest.

## Supplementary Material

FIGURE S1 Original raw images of **Figure 4A**. Validation of transgenic plants. PCR analysis of genomic DNA extracted from hygromycinresistant regenerants.

TABLE S1 Adaptor and primer sequences used.

TABLE S2 Selected metabolites identified by GC-MS in roots of Oe1 under LP-treated conditions compared with WT.

TABLE S3 Selected metabolites identified by GC-MS in the shoots of Oe1 under LP-treated conditions compared with WT.

TABLE S4 Significant pathways identified by KEGG analysis.

TABLE S5 Pathways involved in the roots of Oe1 under LP-treated conditions 0 day compared with WT identified by KEGG analysis.

TABLE S6 Pathways involved in the roots of Oe1 under LP-treated conditions 2 days compared with WT identified by KEGG analysis.

TABLE S7 Pathways involved in the roots of Oe1 under LP-treated conditions 7 days compared with WT identified by KEGG analysis.

TABLE S8 Pathways involved in the shoots of Oe1 under LP-treated conditions 0 day compared with WT identified by KEGG analysis.

TABLE S9 Pathways involved in the shoots of Oe1 under LP-treated conditions 2 days compared with WT identified by KEGG analysis.

TABLE S10 Pathways involved in the shoots of Oe1 under LP-treated conditions 7 days compared with WT identified by KEGG analysis.
