# Supplementary figures and images for: Overexpression of Phosphate Transporter Gene CmPht1;2 Facilitated Pi Uptake and Alternated the Metabolic Profiles of Chrysanthemum Under Phosphate Deficiency
Source: Front Plant Sci. 2018 Jul 20;9:686. doi: 10.3389/fpls.2018.00686 (PMC6062769; doi:10.3389/fpls.2018.00686)

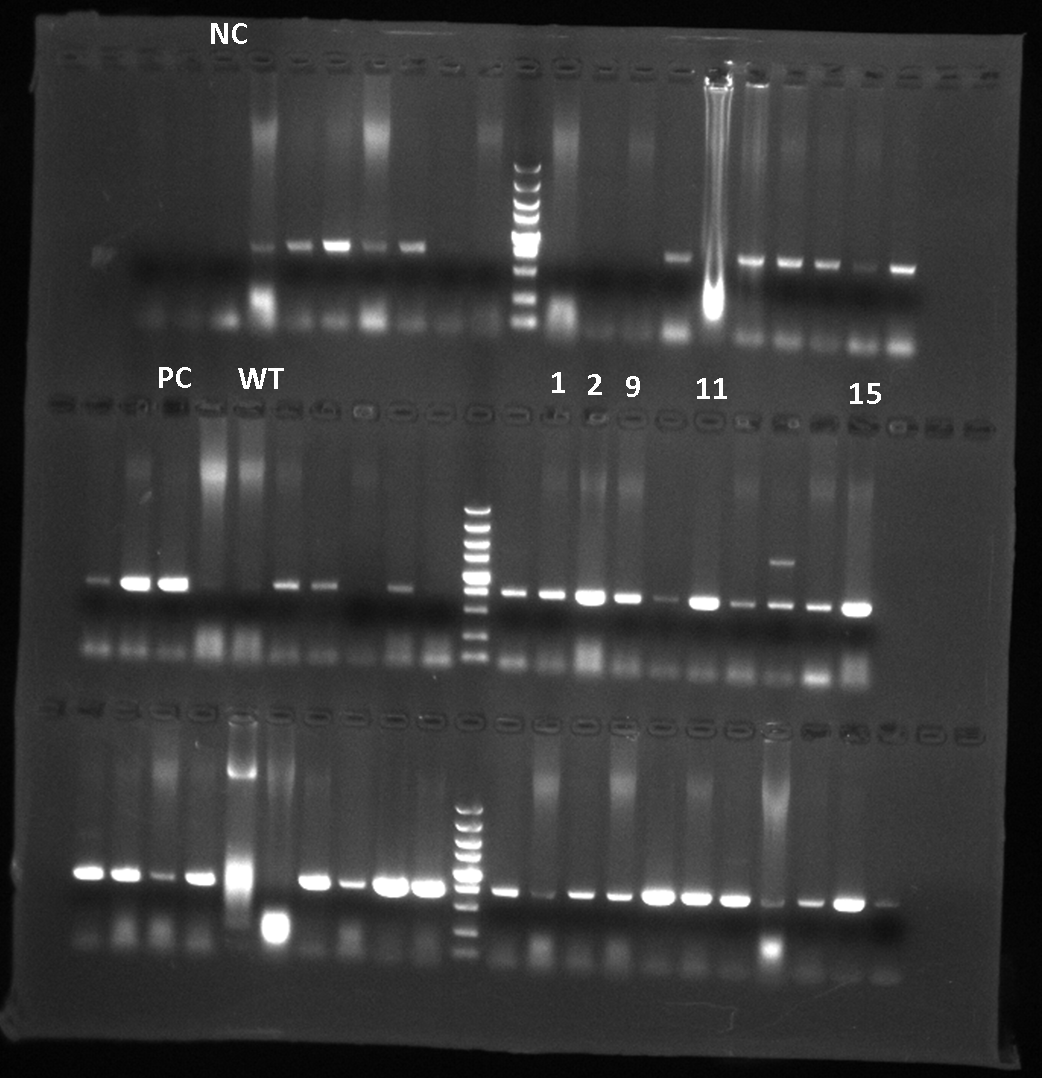

Supplement: FIGURE S1 — Original raw images of Figure 4A. Validation of transgenic plants. PCR analysis of genomic DNA extracted from hygromycinresistant regenerants. [file Image_1.TIF]
